# Chronically shortened rod outer segments accompany photoreceptor cell death in Choroideremia

**DOI:** 10.1371/journal.pone.0242284

**Published:** 2020-11-17

**Authors:** Ingrid P. Meschede, Thomas Burgoyne, Tanya Tolmachova, Miguel C. Seabra, Clare E. Futter

**Affiliations:** 1 UCL Institute of Ophthalmology, London, United Kingdom; 2 Imperial College London, London, United Kingdom; 3 CEDOC, NOVA Universidade Nova de Lisboa, Lisbon, Portugal; University of Florida, UNITED STATES

## Abstract

X-linked choroideremia (CHM) is a disease characterized by gradual retinal degeneration caused by loss of the Rab Escort Protein, REP1. Despite partial compensation by REP2 the disease is characterized by prenylation defects in multiple members of the Rab protein family that are master regulators of membrane traffic. Remarkably, the eye is the only organ affected in CHM patients, possibly because of the huge membrane traffic burden of the post mitotic photoreceptors, which synthesise outer segments, and the adjacent retinal pigment epithelium that degrades the spent portions each day. In this study, we aimed to identify defects in membrane traffic that might lead to photoreceptor cell death in CHM. In a heterozygous null female mouse model of CHM (*Chm*^*null/WT*^), degeneration of the photoreceptor layer was clearly evident from increased numbers of TUNEL positive cells compared to age matched controls, small numbers of cells exhibiting signs of mitochondrial stress and greatly increased microglial infiltration. However, most rod photoreceptors exhibited remarkably normal morphology with well-formed outer segments and no discernible accumulation of transport vesicles in the inner segment. The major evidence of membrane trafficking defects was a shortening of rod outer segments that was evident at 2 months of age but remained constant over the period during which the cells die. A decrease in rhodopsin density found in the outer segment may underlie the outer segment shortening but does not lead to rhodopsin accumulation in the inner segment. Our data argue against defects in rhodopsin transport or outer segment renewal as triggers of cell death in CHM.

## Introduction

The inherited retinal degenerative diseases that are collectively known as retinitis pigmentosa (RP) affect about 1 in 4000 people and are characterized by progressive photoreceptor degeneration leading to visual loss and in some cases blindness [1]. Although causative mutations in about 100 genes have been identified [1], it remains unclear why these lead to photoreceptor death. Some of these mutations affect traffic of components of the phototransduction machinery, such as rhodopsin, either through mutation of the protein itself or of components of the trafficking machinery, raising the possibility that trafficking defects could underlie photoreceptor cell death in some of the most common types of RP. The post mitotic photoreceptors are subject to photo-oxidative stress and so their outer segments (OS) that contain the phototransduction machinery constantly need to be replaced. Every day, the distal 10% of the photoreceptor OS are phagocytosed by the adjacent retinal pigment epithelium (RPE) and replaced proximally by newly synthesized discs [2,3]. Transport of newly synthesised OS components from the inner segment (IS) through the connecting cilium to the OS, their assembly into discs, and the degradation of the phagocytosed OS by the RPE places a huge membrane traffic burden on both photoreceptors and RPE. In the RPE there is a gradual accumulation of intracellular deposits with age, believed, at least in part, to derive from a failure to fully degrade the products of daily phagocytosis of OS [4], which likely compromises RPE health and is particularly marked in age-related macular disease (AMD) (and other diseases). The effect of the daily membrane traffic burden on the health of the photoreceptors is less clear.

To investigate the relationship between membrane traffic defects and photoreceptor cell death, we have taken an RP-related disease whose primary cause is membrane traffic dysfunction. X-linked choroideremia (CHM) is characterized by gradual degeneration of the RPE, photoreceptors and choriocapillaris and is caused by loss of function of Rab Escort Protein-1 (REP1) [5]. In the UK, we estimate a prevalence of 1 in 110,000 for CHM based on the comprehensive study of Pontikos et al [6]. REP1 presents newly synthesized Rab proteins to geranylgeranyl transferase to allow Rab prenylation, a lipid modification essential for Rab membrane association and, hence, Rab function. Rab proteins are low molecular weight GTPases of the RAS superfamily that, in their GTP bound form, undergo prenylation-dependent membrane association where they recruit multiple effectors that regulate interaction with the cytoskeleton, membrane targeting and membrane fusion [7]. More than 60 Rabs in the human genome demarcate overlapping membrane domains and are ‘master regulators of membrane traffic’. In mammals, REP2 partially compensates for loss of REP1 function [8,9]. However, a subset of Rabs are poorly prenylated in CHM [10,11]. No Rab is completely unprenylated in this disease and so no membrane traffic pathway is completely dysfunctional which has made it difficult to establish the underlying causes of photoreceptor and RPE cell death in this disease.

The close relationship between the RPE and photoreceptors raises the possibility that photoreceptor cell death could be secondary to defects in the RPE. However analysis of a photoreceptor-restricted knock-out of REP1 (*Chm*^*Flox*^, *IRBP-Cre*+), led us to conclude that cell death arises autonomously in photoreceptors although photoreceptor cell death was accelerated when REP1 was also lost in the RPE [12].

Although defects in secretion of cytokines can be detected in peripheral blood cells from CHM patients [13], CHM is primarily an ocular disease, indicating either that one or more of the under prenylated Rabs in CHM is of particular importance to the retina or that the retina is peculiarly sensitive to partial defects in membrane traffic. Two of the most affected Rabs in CHM are Rab27a [10,11] and Rab38 [11], but loss of function of these Rabs individually does not lead to the retinal degeneration characteristic of our CHM models [14,15]. Although there are other Rabs affected in CHM, one or more of which could have a particularly important role in the retina, we have found evidence that, at least in the RPE, the traffic burden of these cells could render them peculiarly sensitive to partial membrane traffic defects [16]. When REP1 function is lost in the RPE phagosome degradation is delayed, and this likely contributes to the age-dependent patchy accumulation of intracellular and extracellular deposits and thickening of Bruch’s membrane that occurs in this CHM model [16]. In the current study, we aimed to identify defective membrane traffic pathways caused by loss of REP1 in the photoreceptors, focusing on the daily biogenesis of rod OS. The >2000 molecules/minute of rhodopsin that traffic from the IS to the OS to support this biogenesis makes this transport pathway one potentially sensitive to a partial loss of efficiency. Furthermore, the post Golgi trafficking of rhodopsin is known to depend on the function of multiple Rab proteins [17] and rhodopsin mislocalisation caused by rhodopsin mutation in RP [18], or by loss of function of kinesin II [19], are associated with rapid photoreceptor cell death. We find that from a young age the OS are shorter in heterozygous null females (*Chm*^*null/WT*^). Their length remains relatively constant from 2–12 months, during which time the majority of photoreceptor death occurs, suggesting that OS shortening is due to defect(s) in membrane traffic pathways, rather than a consequence of retinal degeneration. Despite the shortening, OS structure is largely normal and is not accompanied by accumulation of rhodopsin or transport vesicles in the IS. Our data therefore suggest that, although defects in OS degradation may contribute to RPE cell degeneration, defects in OS biogenesis are not a direct cause of photoreceptor cell death in CHM.

## Material and methods

### Animal care

Animals used in this study were treated in accordance with UK Home Office regulations under project licences 70/6176 and 70/7078 and in strict agreement with the Association for Research in Vision and Ophthalmology (ARVO) Statement for the Use of Animals in Ophthalmic and Vision Research. Animal experiments were approved by the Imperial College's Animal Welfare and Ethical Review Body (AWERB). Mice were housed in individually ventilated cages on 12-hour light/dark cycle with free access to food and water. All animals were sacrificed by dislocation of the neck; death was confirmed by the cessation of the blood circulation. In accordance with the Home Office guidance, no anaesthesia was required.

### Mouse strains

Majority of experiments were performed using heterozygous null female mouse model of CHM (*Chm*^*null/WT*^) that was described previously [20] and age- and sex-matched controls (*Chm* ^*WT/WT*^). Mice carrying conditional *Chm* alleles (*Chm* ^*Flox*^ and *Chm* ^*3lox*^) with or without tamoxifen (TM)-inducible MerCreMer (MCM) transgene were described previously [20]. Animals with conditional alleles were used mainly as controls in biochemical studies in minimal numbers (3–5 animals per strain). To generate *Chm*^*null/WT*^ females in this study we devised a new breeding scheme.*Chm* ^*Flox*^*/ Y* males were crossed with *CHM*^*WT/WT*^ females carrying PGK-Cre transgene, which causes early and uniformal rearrangement of *Chm*^*Flox*^ allele into *Chm*^*null*^ allele. Rearrangement was confirmed by genotyping as described in [20]. Both parental strains have no adverse ill effects.

### Antibodies and reagents

Microglia Iba-1 specific antibody was from MenaPath/A. Menarini Diagnostics Ltd (Winnersh, **UK)**; anti-rhodopsin against the C-terminus (1D4) and N-terminus (RetP1) were from Abcam (Cambridge-UK), secondary antibody conjugated to Alexa Fluor 488 from Molecular Probes (Eugene, USA); rabbit anti-mouse bridging antibody was from Dako Ltd. (Ely, UK) and protein-A-gold from University Medical Center (Utrecht-NL). J905 is a rabbit polyclonal antibody directed against recombinant rat *REP-1* which recognises both REP1 and REP2 [20].

### Tunel assay

Photoreceptor apoptosis was determined by terminal deoxynucleotide transferase nick-end labelling (TUNEL) assay using the *In Situ* Cell Death Detection Kit, TMR Red (cat. number 12156792910; Roche Applied Science, Penzberg, Germany). Mouse eyes were fixed for 1h in 4% (wt/vol) paraforaldehyde (PFA) in PBS. The eyes were cut along the ora serrata and the lens and cornea were removed. Five radial cuts were made to open the eyecup. The neuroretina was gently peeled, fixed for another hour in 4% (wt/vol) PFA in PBS, washed three times in 2X PBS, permeabilised overnight with 3% (vol/vol) TritonX-100 and 0.1% (vol/vol) Tween20 in 2X PBS and TUNEL staining was performed according to the manufacturer’s instructions, except that incubation with TUNEL reaction mixture was for 4h at room temperature. An internal positive control was used where neuroretinas were pre-treated with DNAse after permeabilisation. All specimens were examined on a Zeiss LSM 710 confocal microscope (Carl Zeiss Meditec AG, Jena, Germany). TUNEL positive cells/mm^2^ were quantified by ImageJ.

### Immunofluorescence analysis

Mouse eyes were fixed in 4% (wt/vol) PFA in PBS for 2 hours at room temperature. For OCT sections, eyes were infiltrated overnight in 30% (wt/vol) sucrose in PBS at 4° C and embedded in Tissue Tek® O.C.T^TM^. 12-μm thick transverse sections, adjacent to the optic nerve, were cut at −20°C and dried at room temperature. Sections were immunolabelled with anti-Rhodopsin RetP1 antibody overnight at 4° C, followed by secondary antibody conjugated to Alexa Fluor 488 and DAPI for 1h at room temperature. Sections were imaged and analysed using a Zeiss confocal LSM 710 microscope system (Carl Zeiss Meditec AG, Jena, Germany). For whole mount microglia Iba-1 staining, after fixation, the eyes were cut along the ora serrata, cornea and lens were removed, and fixed for another hour at room temperature. Five radial cuts were made to open the eye cup and neuroretina was gently peeled off. Eye cups were washed three times in 2X PBS, incubated for 2h at room temperature with blocking buffer (3% (vol/vol) TritonX-100 and 0.1% (vol/vol) Tween20 in 2X PBS) and stained with Iba-1 antibody in blocking buffer overnight followed by 2h incubation with Alexa Fluor 488 secondary antibody and DAPI at room temperature. Whole mounts were imaged using a Zeiss Axiophot microscope (Carl Zeiss Meditec AG, Jena, Germany).

### Light and electron microscopy of eye cup sections

Mouse eyes were fixed for 1.5h in 2% (wt/vol) PFA, 2% (vol/vol) glutaraldehyde in 0.1M cacodylate buffer. The cornea and lens were removed, and the eye cup was postfixed in 1% (wt/vol) osmium tetroxide, 1.5% (wt/vol) potassium ferrocyanide in 0.1M cacodylate at for 2h at 4°C. Eye were dehydrated using increasing concentrations of ethanol (70%, 90% and absolute) and propylene oxide, transferred to 1:1 propylene oxide:Epon overnight, followed by two changes of Epon alone before embedding in Epon resin.

To measure OS length, semi-thin 0.75μm sections were cut, collected on glass slides and dried on a hot plate. Sections were stained with 1% (wt/vol) toluidine blue in 1% (wt/vol) sodium borate for 30s on a hot plate, quickly washed with a stream of distilled water, rinsed with 50% ethanol and left to dry on a hot plate. Samples were analysed using a Zeiss LSM 510 microscope (Carl Zeiss Meditec AG, Jena, Germany).

To examine photoreceptor ultrastructure and measure OS width, ultra-thin 70-80nm sections were cut, collected on Formvar/carbon coated slot grids and stained with lead citrate before examination on a JEOL 1010 transmission electron microscope (Welwyn Garden City, UK). Images were taken with a Gatan Orius SC1000B charge-coupled device camera and analysed with Gatan Digital Micrograph (Pleasanton, USA).

### Immunoblotting and *in vitro* prenylation assays

Immunoblotting of mouse tissues was performed as previously described [21] using an anti-REP antibody (J905) which recognises both REP1 and REP2 proteins. The *in vitro* prenylation assay was performed as previously described [12].

### Cryo-immuno electron microscopy

Mouse eyes were fixed in 4% PFA (wt/vol) in 0.1 M phosphate buffer for 3h, the cornea was cut off and lens removed. The retina was cut into small pieces, embedded in 12% (wt/vol) gelatine and infused with 2.3 M sucrose overnight at 4°C. 70-90nm sections were cut at −120°C and collected in 1:1 mixture of 2.3 M sucrose: 2% (wt/vol) methylcellulose. Sequential double labelling with different sizes of gold particles was done as previously [22]. Sections were labelled with antibody against the C-terminus of rhodopsin (1D4) followed by a rabbit-anti-mouse bridging antibody and protein A gold and then a second round of staining with antibody against the N-terminus of rhodopsin (RetP1). Samples were analysed with a JEOL 1010 transmission electron microscope and imaged using Gatan Orius SC1000B charge-coupled device camera.

### Quantification

For quantification of TUNEL positive cells, the area of entire neuroretinal whole mount was analysed for TUNEL positive cells and the number of cells was divided by the area the whole mount.

For mitochondrial analysis, low magnification electron microscopy images scanning whole retina (periphery-central-periphery) were taken and number of photoreceptors containing swollen mitochondria and damaged OSs were manually counted and divided by the total length of the retina. For Iba-1 positive cells quantification, the number of positive cells were counted per eye cup under the microscope.

For analysis of OS length, low magnification images of the whole length of the retina were montaged and divided into 10 regular intervals (from one periphery to the other) and the length of the OS was measured in each. The width of the OS was measured from EM images at random regions along the length of the retina.

For rhodopsin quantification of immunoEM images, the inner segment was divided in 3 equal regions, basal (containing the Golgi), middle and apical, using an ImageJ macro. Gold particles/unit area were counted in the distal third of the OSs and in each region in the inner segment.

## Results

### Small numbers of photoreceptors in *Chm*^*null/WT*^ exhibit signs of apoptosis, and mitochondrial stress, accompanied by infiltration of inflammatory cells

**Fig 1:**
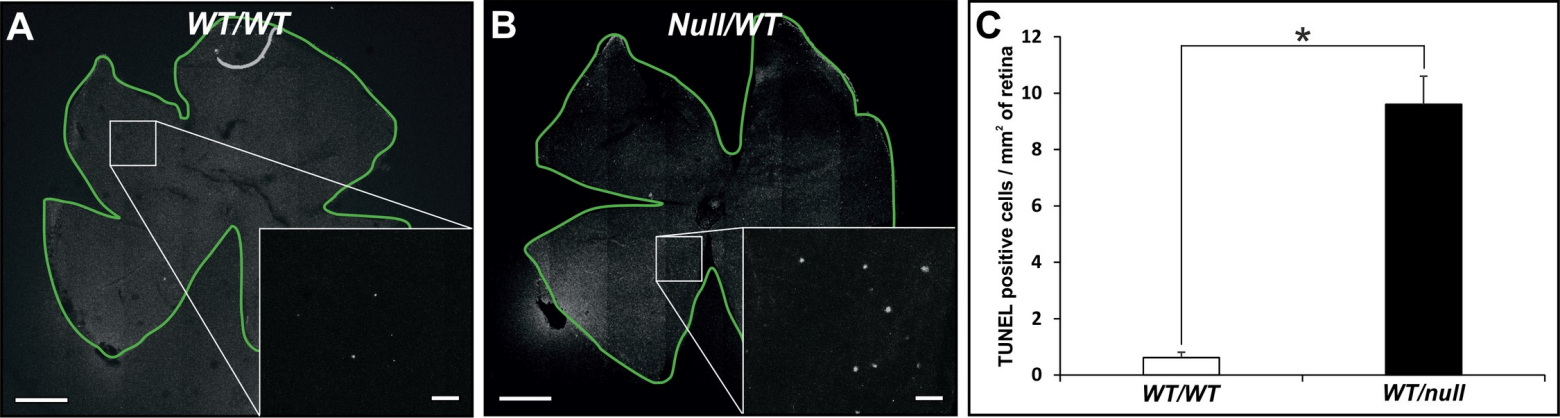
Elevated numbers of TUNEL positive cells in *Chm*^*null/WT*^. (A, B) TUNEL assays were performed on flat mounted entire retinae from 6- month-old heterozygous null female mice (*Chm*^*null/WT*^) and age-matched controls. The boxed region is magnified in the inset to reveal individual nuclei. Scale bars: 500μm (A, B) and 20μm (insets). C: TUNEL positive cells/mm^2^ of retina were quantitated. T test: *p< 0.05, n = 3.

**Fig 2:**
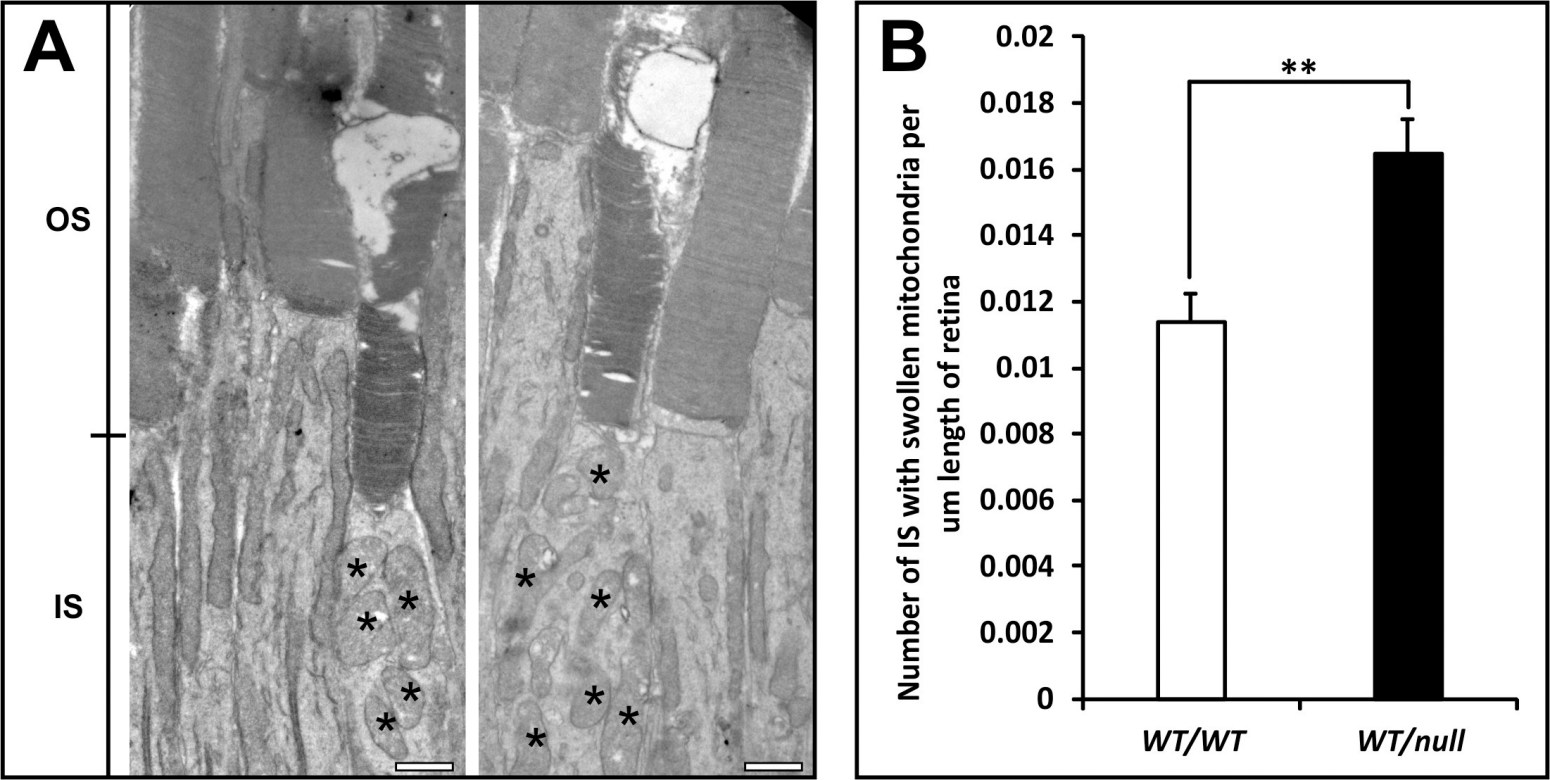
Elevated numbers of rods with swollen mitochondria in *Chm*^*null/WT*^. Eyes from 6-month-old heterozygous null female mice (*Chm*^*null/WT*^) were processed for transmission electron microscopy. (A) Analysis along the full length of the retina (from peripheral through central to peripheral) revealed occasional cells with swollen mitochondria and degenerating outer segments (OS) in both *Chm*^*null/WT*^ and controls. Scale bars: 1μm. (B) Quantitative analysis revealed a greater number of such cells in *Chm*^*null/WT*^. T test: **p<0.01, n = 8.

**Fig 3:**
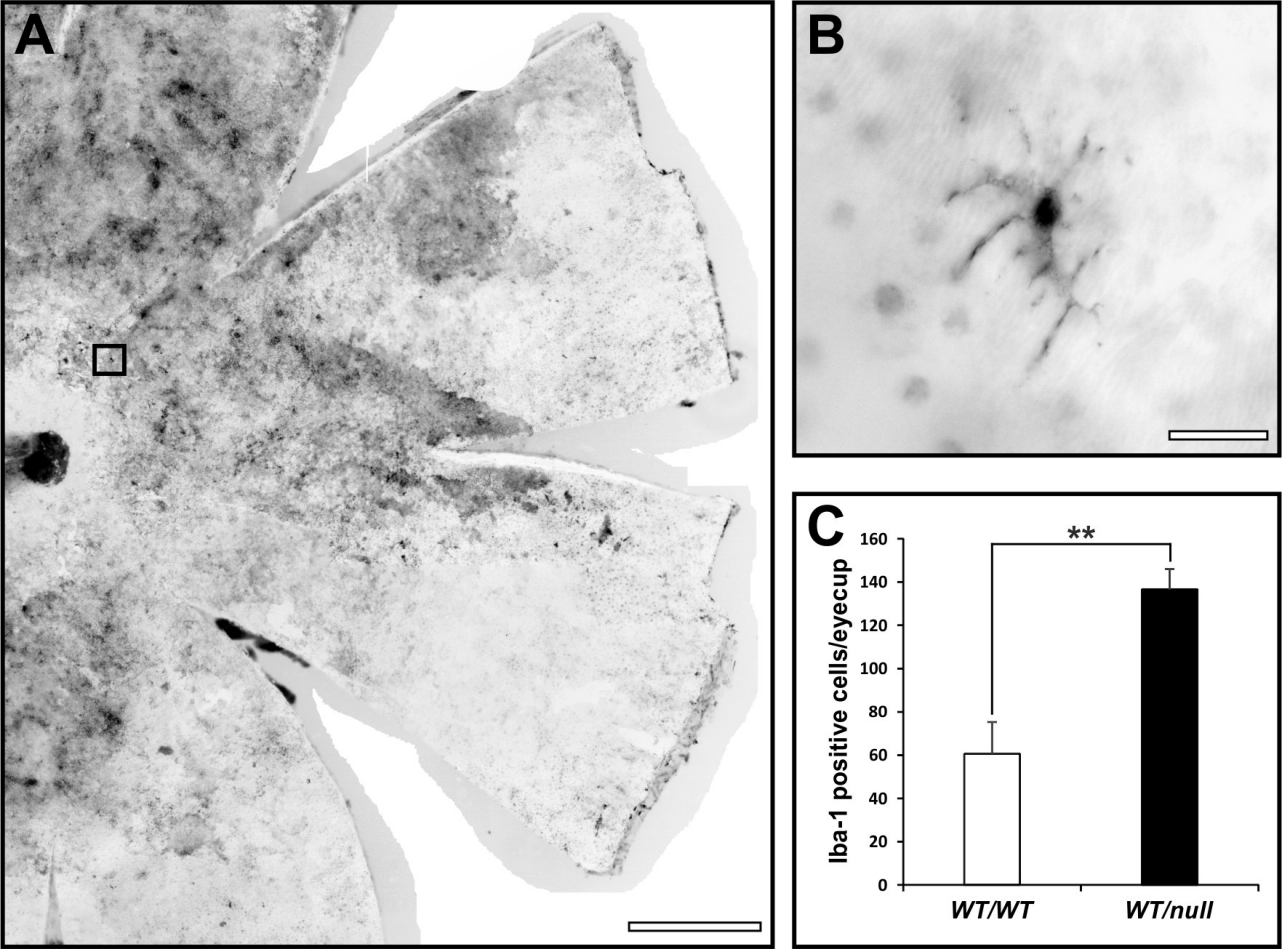
Elevated numbers of Iba1 positive glia in *Chm*^*null/WT*^. (A) Flat mounted entire retinae from 6-month-old heterozygous null female mice were stained for the glial cell marker, Iba1. (B) High magnification of the boxed region in A indicates the stellate morphology of the glial cells. Scale bars: 500 μm (A) and 100μm (B). (C) Quantitation of glial cell numbers in heterozygous null female mice and age-matched controls reveals elevated numbers in *Chm*^*null/WT*^. T-test: **p<0.01, n = 3.

We have generated multiple mouse models of CHM but in this manuscript have focused on heterozygous null females (*Chm*^*null/WT*^) that show the maximum rate of photoreceptor degeneration, as indicated by loss of photoreceptor nuclei. In this model there is a gradual loss of photoreceptors over >12 months. Apoptosis has been extremely difficult to detect in models of CHM, most likely because of the slow rate of cell death meaning that very few cells are undergoing apoptosis at any one time. We found that to detect significant numbers of TUNEL positive cells it was necessary to develop a flat mount assay that allowed TUNEL positive photoreceptors to be quantitated on the entire retina of single mouse eyes. This analysis revealed a 15.5-fold increase in numbers of TUNEL positive cells in *Chm*^*null/WT*^ at 6 months of age, compared with age-matched controls (Fig 1). Although most photoreceptors at this age in *Chm*^*null/WT*^ exhibit normal gross morphology, a small subpopulation exhibited swollen mitochondria (Fig 2), a recognized sign of mitochondrial stress that could lead to cell death. Consistently, the majority of cells with swollen mitochondria also exhibited degenerating OS, although it was not possible to be sure that all cells with swollen mitochondria were degenerating as the entire photoreceptor was not always in the plane of section. We have previously noted increased numbers of Iba1 positive glial cells between the RPE and photoreceptors in tissue-restricted mouse models of CHM, with the highest levels of glial infiltration being seen accompanying the highest levels of photoreceptor death [12]. Consistently we observed greatly elevated levels of glial infiltration in retinae from *Chm*^*null/WT*^ (Fig 3).

### The majority of photoreceptors in *Chm*^*null/WT*^ have normal ultrastructure

**Fig 4:**
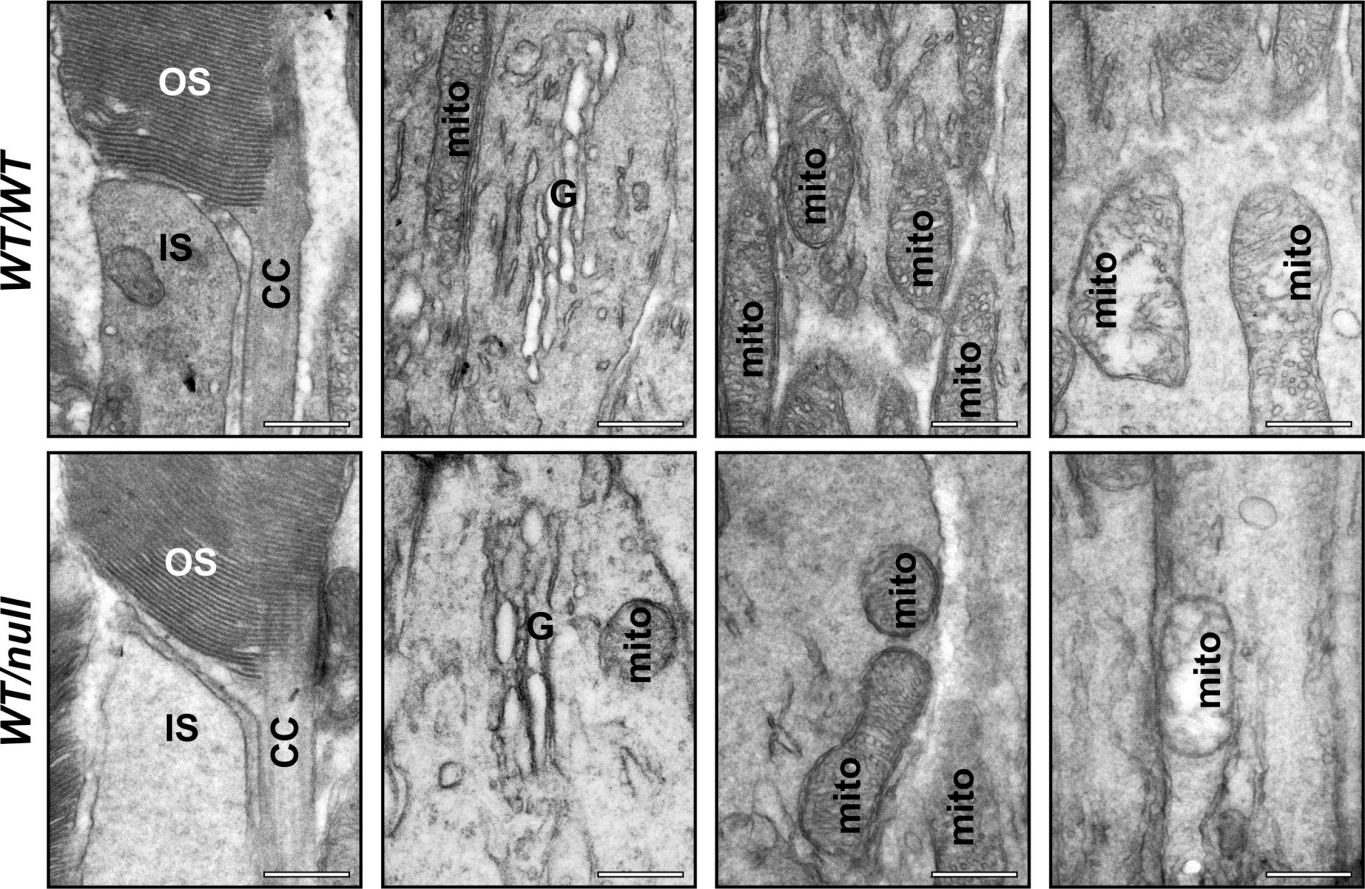
*Chm*^*null/WT*^ show normal ultrastructure of the inner segment, outer segment and connecting cilium. Eyes from 6-month-old heterozygous null female mice (*Chm*^*null/WT*^) were processed for transmission electron microscopy. High magnification examination of longitudinal sections through the eye cup revealed no abnormalities of the outer segment structure (OS) or connecting cilium (CC) or the Golgi apparatus (G) or mitochondria (mito) in the inner segment (IS) in the majority of cells. Scale bars: 500nm.

The daily requirement for the multistep process of generating OS components within the IS, transporting them through the connecting cilium to the OS and assembling the OS discs, led us to hypothesise that chronic partial defects in the membrane traffic pathways that underlie OS biogenesis might lead to morphologically identifiable defects in OS structure. However, the structure of the OSs in the majority of *Chm*^*null/WT*^ photoreceptors was indistinguishable from age-matched controls (Fig 4). Most OS contained well-ordered stacks of discs. Furthermore, detailed examination of the IS did not reveal any build-up of transport vesicles and the structure of the connecting cilium was indistinguishable from age-matched controls (Fig 4).

### Shortened photoreceptor outer segments are a constant uniform feature of *Chm*^*null/WT*^

**Fig 5:**
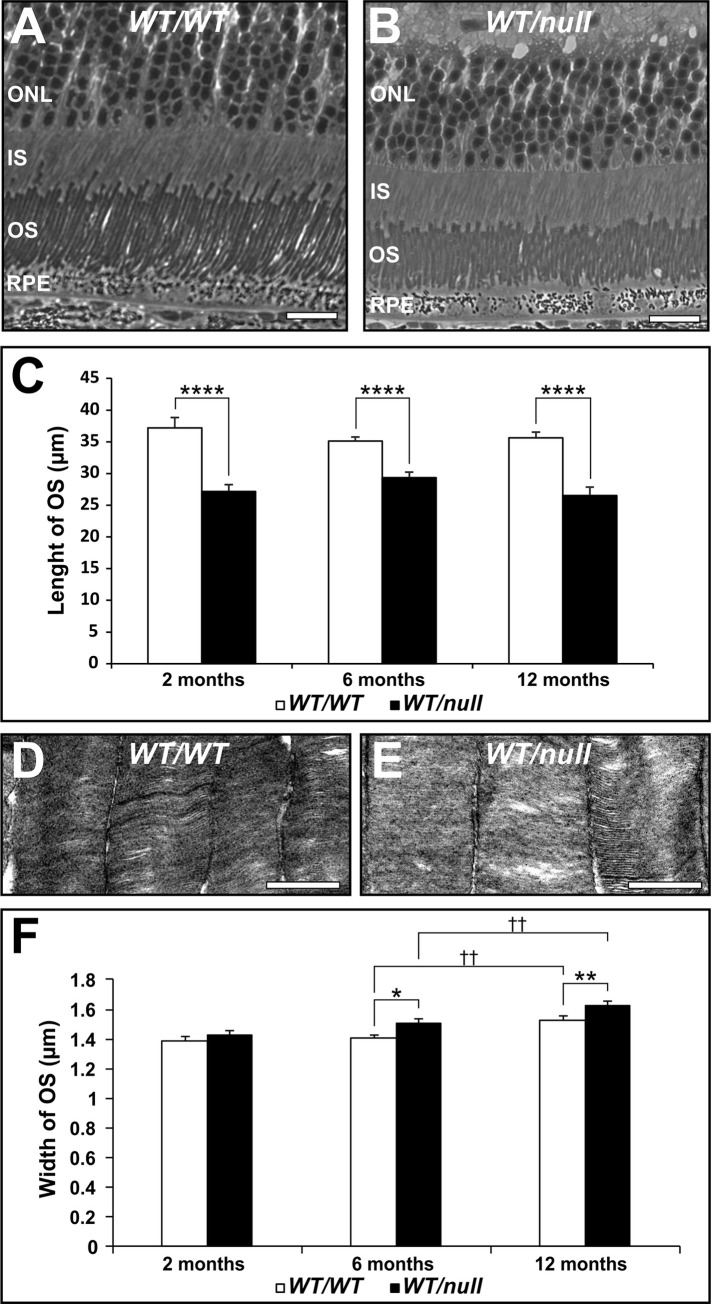
*Chm*^*null/WT*^ have uniformly shortened outer segments. Eyes from 2, 6 and 12 month-old heterozygous null female mice (*Chm*^*null/WT*^) were processed for transmission electron microscopy. Low magnification examination of longitudinal sections throughout the length of the retina (from peripheral through central to peripheral), at 10 uniform intervals revealed uniform shortening of the OS (A-C). Higher magnification images revealed progressive increases in OS width (D-F). Scale bars: 20 μm (A,B), 1μm (D,E). T-test: ****p<0.001, n = 6 (C), *p<0.05, **p<0.01, †† p<0.01 n = 3 (F).

Although the ultrastructure of most OS was normal, most OS in *Chm*^*null/WT*^ were shorter than controls (Fig 5A–5C). Even though CHM is a progressive retinal degenerative disease, the rab prenylation defects that lead to partial dysfunction in membrane traffic pathways in this disease are present throughout life. Thus, if the shortening of photoreceptor OS is due to partial defects in membrane traffic pathways, the OS shortening should be present from an early age and remain constant. Accurate measurement of OS length indeed indicates a 27% shortening of OS that is detectable within the first 2 months of life and remains fairly constant for at least the next 10 months (Fig 5C), during which the majority of photoreceptor cell death occurs in this model [20]. Interestingly, OS length is fairly even across the retina despite this being a heterozygous model. Western blotting indicated that the amount of REP1 protein was reduced by approximately 50% in tissues from 6–8 month old mice (S1 Fig). Reduced levels of REP1 protein would be expected to result in reduced Rab prenylation. This can be tested by performing *in vitro* Rab prenylation assays as only underprenylated Rabs are available for *in vitro* prenylation. *In vitro* prenylation was virtually undetectable in the presence of wild type levels of REP1 (S2 Fig). Consistent with a partial loss of REP1 protein, there was a higher level of *in vitro* Rab prenylation in *Chm*^*null/WT*^ but not as high as in a tamoxifen-induced knockout of REP1. There was a small but progressive increase in OS width in *Chm*^*null/WT*^ compared to controls (Fig 5D–5F), which could be due to reduced spatial constraints as photoreceptors die.

### Lower density of rhodopsin in the inner and outer segments without accumulation of rhodopsin in the inner segment of *Chm*^*null/WT*^

**Fig 6:**
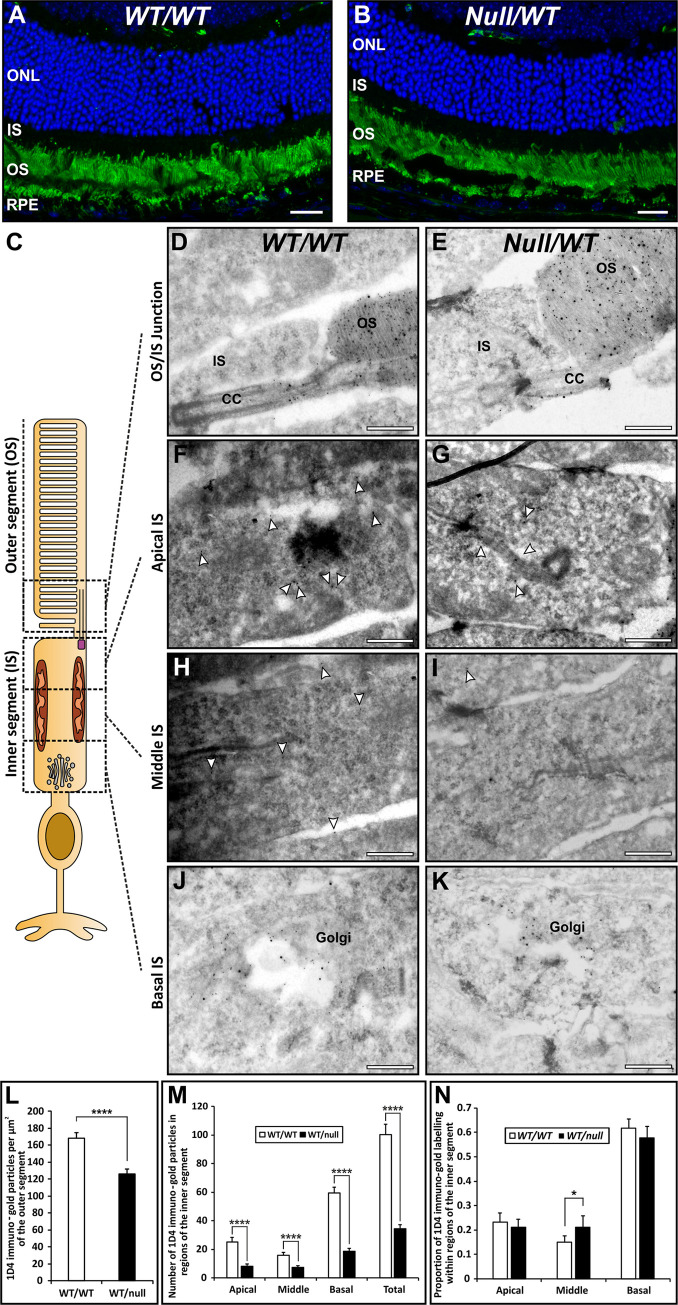
Reduced rhodopsin in the outer segment is not accompanied by accumulation in the inner segment. (A, B) Rhodopsin staining (green) of 12μm retinal sections of 6-month-old heterozygous null female mice (*Chm*^*null/WT*^) revealed strongly positive outer segments (OS), a small number of rhodopsin positive punctae (phagosomes) in the retinal pigment epithelium (RPE) but no detectable staining in the inner segment (IS). This was despite evident photoreceptor loss, as indicated by reduced numbers of DAPI stained (blue) nuclei in the outer nuclear layer (ONL). (C-K) Eye cup-derived specimens from 6-month-old heterozygous null female mice (*Chm*^*null/WT*^) and age-matched controls were stained for rhodopsin by cryo-immunoEM using antibodies to the C-terminus (1D4-5nm gold) and N-terminus (RetP1-15nm gold). Rhodopsin was concentrated on the outer segment (OS) discs and the limiting membrane of the connecting cilium (CC). Rhodopsin labelling (examples indicated by white arrowheads in F-I) was also present on the Golgi and on small vesicles within the inner segment. Scale bars: 20 μm (A,B) and 200nm (D-K). (L,M) Quantitation of the number of 1D4 rhodopsin gold particles/area of IS and OS revealed that rhodopsin concentration was reduced to a similar extent in IS and OS of heterozygous null female mice (*Chm*^*null/WT*^). (N) Dividing the IS into equal basal (containing the Golgi), middle and apical domains revealed a shift in the distribution of gold particles in the IS to a greater proportion in the middle region (mainly vesicle-associated). T-test: *p<0.05, ****p<0.0001, n = 3.

Rhodopsin staining of retinal sections by immunofluorescence revealed strong staining of the OS but no detectable staining in the IS of either *Chm*^*null/WT*^ or controls (Fig 6A and 6B). However, the huge levels of rhodopsin expression could mask subtle changes in rhodopsin transport and so rhodopsin localization was examined by cryo-immunoEM, taking advantage of the high rate of rhodopsin synthesis that allows the biosynthetic pathway to be readily labelled and quantified with rhodopsin immunogold. Quantitation of the density of rhodopsin gold particles on the OS revealed a 25% reduction in rhodopsin density in *Chm*^*null/WT*^ (Fig 6C–6E and 6L), suggesting reduced rhodopsin synthesis and/or transport to the OS. Defective transport between the IS and OS would be expected to lead to accumulation of rhodopsin in the IS. However, photoreceptors of *Chm*^*null/WT*^ exhibited a lower level of rhodopsin staining in the IS than controls (Fig 6C, 6F–6K, 6M and 6N). Quantitating the distribution of rhodopsin within the IS showed, in *Chm*^*null/WT*^, a small reduction in % of rhodopsin in the basal region, that contains the majority of Golgi-associated rhodopsin, but an increase in % of rhodopsin in the middle region that contains the majority of transport vesicles that shuttle rhodopsin from the Golgi to the apical IS plasma membrane (Fig 6M and 6N). Thus, subtle defects in rhodopsin synthesis and transport could contribute to the shortening of photoreceptor OS but do not lead to accumulation of rhodopsin in the IS.

## Discussion

In this study we searched for evidence of defects in membrane traffic pathways that might lead to photoreceptor cell death, using a heterozygous mouse model of choroideremia (*Chm*^*null/WT*^) that shows the maximum rate of cell death of all our CHM mouse models [20]. The rate of cell death is relatively slow, occurring from 2 to >12 months, making it difficult to identify dying cells at any single time point in retinal sections. By analyzing entire retinae, we were able to demonstrate an increase in number of photoreceptors exhibiting DNA fragmentation in our CHM model, as demonstrated by TUNEL staining. DNA fragmentation is a comparatively late and short stage in cell death and does not distinguish between caspase-dependent, caspase-independent, autophagic and necrotic cell death mechanisms, all of which have been implicated in photoreceptor degeneration in RP. Electron microscopic analysis allowed us to detect a small but significantly increased number of photoreceptors with swollen mitochondria in *Chm*^*null/WT*^. Mitochondrial swelling is caused by a loss of control of permeability of the inner mitochondrial membrane and can lead to apoptosis through release of cytochrome C or to necrosis when ATP levels are low. That we could identify these cells in thin sections indicates that this is a more prolonged stage in the degenerative process than DNA fragmentation, but the fragmented OS that frequently accompanied mitochondrial swelling indicates that the latter is a marker of dying cells. One of the most striking features of the photoreceptor cell layer in our CHM model was the rise in the number of infiltrating glia. Glial cell infiltration of the outer retina has been observed in multiple models of RP [23–25] and has been reported to precede or co-incide with the onset of cell death. Although these cells are phagocytic and so could have the beneficial effect of clearing debris from apoptotic cells, they may also themselves promote photoreceptor degeneration through the production of inflammatory cytokines and chemokines [26,27], through the overproduction of reactive oxygen species (ROS) or even through the phagocytosis of living photoreceptors [28]. Consistent with these observations, a mild T-lymphocytic infiltration was observed in a human post-mortem eye [29].

Although the final mechanism(s) of photoreceptor cell death in CHM has yet to be established, it is caused by loss of REP1, implying defective membrane traffic pathway(s). Furthermore, the fact that REP1 is ubiquitously expressed but photoreceptors (and RPE) are peculiarly affected by its loss, implicates dysfunction in membrane traffic pathway(s) of particular importance to photoreceptors. The daily renewal of POS places a huge and unique traffic burden on the biosynthetic and transport machinery of the photoreceptors. Newly synthesized disc proteins must be trafficked via the Golgi apparatus to the inner segment plasma membrane, before undergoing intraflagellar transport (IFT)-mediated transport via the connecting cilium to the OS. At the base of the OS, new discs are formed by evagination of the plasma membrane followed by fusion of the rims of the evaginations [30–33]. We initially focused on OS disc structure as disc disorganization has been described in other models of retinal degeneration, including in heterozygous knock in mice, carrying the most common RP-causing mutation in rhodopsin, P23H [34]. In these mice disc disorganization, including sagitally organised discs, has been observed which precedes retinal degeneration and has been proposed to be a trigger of photoreceptor death, although ER stress caused by aberrant folding of P23H rhodopsin and consequent saturation of the proteosomal system is another proposed cause [35]. Our analysis of *Chm*^*null/WT*^ indicates well-ordered photoreceptor discs, except in the small number of cells with swollen mitochondria and degenerating OS, indicating likely dying cells. Although subtle defects in the complex process of OS renewal cannot be excluded, we found no evidence for a defect in OS disc assembly or maintenance that could trigger photoreceptor cell death in this model.

Although the morphology of most OS in heterozygous null females was normal, OSs were shortened. Progressive OS shortening has been reported in aging [36] and in various models of RP [37], where it has been linked with loss of photoreceptor cell function. However, the shortening of OS in *Chm*^*null/WT*^ is not progressive, being relatively constant from 2 months of age to 12 months, indicating that the OS shortening reflects a trafficking defect, rather than a gradual degenerative process. We previously found that tissue restricted loss of REP1 in the RPE did not cause shortening of the adjacent wild type photoreceptor OSs [16], suggesting that OS shortening is a photoreceptor cell intrinsic phenomenon. The uniformity of OS shortening across the retina was perhaps surprising given that approximately 50% of photoreceptors in this heterozygous CHM model would initially be expected to express wild type levels of REP1. Although REP1 negative photoreceptors might be expected to die preferentially leading to a higher percentage of wild type cells in older mice, our analysis indicated an approximately 50% reduction of REP1 in the neuroretina, compared to wild type mice, at 6–8 months of age. Each photoreceptor interacts (directly or through the interphotoreceptor matrix) with multiple neighbouring photoreceptors in the intact retina and these interactions may play a role in maintaining a relatively uniform OS length despite genetic heterogeneity within the photoreceptor layer of our mouse model. The increase in OS width that accompanied photoreceptor cell death is consistent with a need to maintain a close association between the membranes of neighbouring OSs.

Rhodopsin makes up 50% of OS protein. Rhodopsin null mice do not develop rod OS [38] but rhodopsin^wt/null^ mice display defects somewhat similar to *Chm*^*null/WT*^, in that they have reduced rhodopsin density in the OS [39] or reduced OS size [38,40], although the latter was more evident in older mice [40]. The lower density of rhodopsin that we found in the OS of *Chm*^*null/WT*^, thus provides a potential explanation for the reduced length of the OS, although as photoreceptors die the increased width of the OS means that changes to total OS volume are small. Defective rhodopsin transport from the IS to the OS has been linked to photoreceptor cell death, possibly due to aberrant G protein signalling via rhodopsin accumulated in the IS. However, we failed to detect rhodopsin accumulation in the IS in *Chm*^*null/WT*^, in fact, rhodopsin levels were reduced in the IS, indicating that in this model the photoreceptors may compensate for a reduction in rhodopsin transport by limiting rhodopsin synthesis or enhancing its degradation.

The lack of rhodopsin accumulation anywhere in the IS prevented the identification of a clear rate limiting step in rhodopsin transport but an increase in the proportion of rhodopsin between the Golgi and the apical region of the IS in *Chm*^*null/WT*^ suggested a possible reduction in the rate of transport of Golgi-derived carriers to the apical plasma membrane. Rabs 6, 8a and 11a, which have all been implicated in traffic of rhodopsin carriers from the Golgi to the IS plasma membrane [17], have not been reported to be majorly affected in CHM [11,22]. However Rabs 8a and 11 appear dispensable for rhodopsin transport in the mouse [41], suggesting some redundancy and/or species differences in Rab requirements.

It is important to note that in this study of mouse retina we have focussed on rod photoreceptors, as cones are sparse in the mouse retina. There is considerable evidence for a role of rod dysfunction in CHM, including the preferential loss of rods in a female CHM carrier [42], early changes in CHM tending to occur in the peripheral more rod-rich regions [43] and spectral domain-optical coherence tomography evidence of rod OS abnormalities, most likely OS shortening, that precede retinal remodelling [44]. That loss of REP1 in only 50% of the cells in our CHM mouse model leads to consistent OS shortening and significant apoptosis in the rod photoreceptor layer supports an important role of rod dysfunction in the human disease.

In summary, neither OS disruption, nor accumulation of rhodopsin in the IS, are likely triggers of cell death in this CHM model. We cannot exclude the possibility that subtle changes in the complex process of OS renewal may occur that are undetectable by the experimental approach used here and REP1 loss in the photoreceptors does lead to OS shortening and reduced density of rhodopsin in the outer segments. Rods appear to compensate for REP1 loss by reducing rhodopsin synthesis or enhancing its degradation, thereby, reducing the burden of OS renewal. Although on its own this is also unlikely to trigger photoreceptor cell death, it could reduce the oxygen consumption of the OS, leading to a potential rise in the pro-oxidative local environment and consequent rise in pro-apoptotic signalling. This may be further exacerbated by ROS production by infiltrating microglia and reduced protection from oxidative stress due to the patchy depigmentation and reduced apical movement of melanosomes in the RPE that have been previously described in this CHM model [20]. Consistent with its high energy demand, the IS is rich in mitochondria, which are both a source and target of ROS. Our finding of swollen mitochondria in photoreceptors that are likely committed to cell death would be consistent with a model whereby shortened OS, infiltrating microglia and reduced melanin protection enhance the photoreceptors exposure to oxidative stresses that lead to mitochondrial dysfunction and increased susceptibility to cell death. It is likely that partial dysfunction of Rabs associated with mitochondrial homeostasis (through the regulation of mitochondrial fission, fusion, movement and formation of contacts with the ER) may make mitochondria within photoreceptors less able to respond to oxidative stress. Perturbations in mitochondrial homeostasis, rather than in OS renewal, may therefore be key to understanding photoreceptor cell death in CHM.

## Supporting information

S1 Checklist The ARRIVE guidelines 2.0: Author checklist. (PDF)

S1 Fig Rep1 expression in tissues of *CHM*^*null/WT*^ females is reduced in comparison to the wild type mice. Protein extracts were isolated from mouse tissues indicated underneath each blot (n = 1), subjected to SDS-PAGE and analysed by immunoblotting using anti-Rep antibody (J905) which recognises both Rep1 and Rep2 (dilution 1:500). Age of animals: 8–9 months. Rep2 band was used as a loading control. Experiment was repeated 3 times.
(PDF)

S2 Fig Pattern of underprenylated Rabs in *CHM*^*null/WT*^ is similar to tamoxifen(TM)-induced *CHM* knockout, yet amount of newly prenylated Rabs in *CHM*^*null/WT*^ is lower, which is consistent with only 50% of cells carrying null allele. *In vitro* prenylation reaction was performed on the cytosolic lysates isolated from the neuroretina and RPE of the *CHM*^*WT/WT*^ (n = 4), *CHM*^*Flox*^*/Y* (n = 5), TM-induced *CHM*^*3lox*^*/Y*, *MerCreMer (MCM)* (n = 3) and *CHM*^*null/WT*^ (n = 5) mice. *CHM*^*Flox*^ is a conditional allele with level of Rep1 similar to *CHM*^*WT*^. *CHM*^*3lox*^ is a conditional hypomorphic allele of *CHM* gene. TM induction of *CHM*^*3lox*^*/Y*, *MCM* leads to *CHM* knockout. Age of animals: 6–8 weeks.
(PDF)
