# Supplementary material for: Chronically shortened rod outer segments accompany photoreceptor cell death in Choroideremia
Source: PLoS One. 2020 Nov 17;15(11):e0242284. doi: 10.1371/journal.pone.0242284 (PMC7671558; doi:10.1371/journal.pone.0242284)
Supplement: S1 Fig — Protein extracts were isolated from mouse tissues indicated underneath each blot (n = 1), subjected to SDS-PAGE and analysed by immunoblotting using anti-Rep antibody (J905) which recognises both Rep1 and Rep2 (dilution 1:500). Age of animals: 8–9 months. Rep2 band was used as a loading control. Experiment was repeated 3 times. (PDF) [file pone.0242284.s002.pdf]

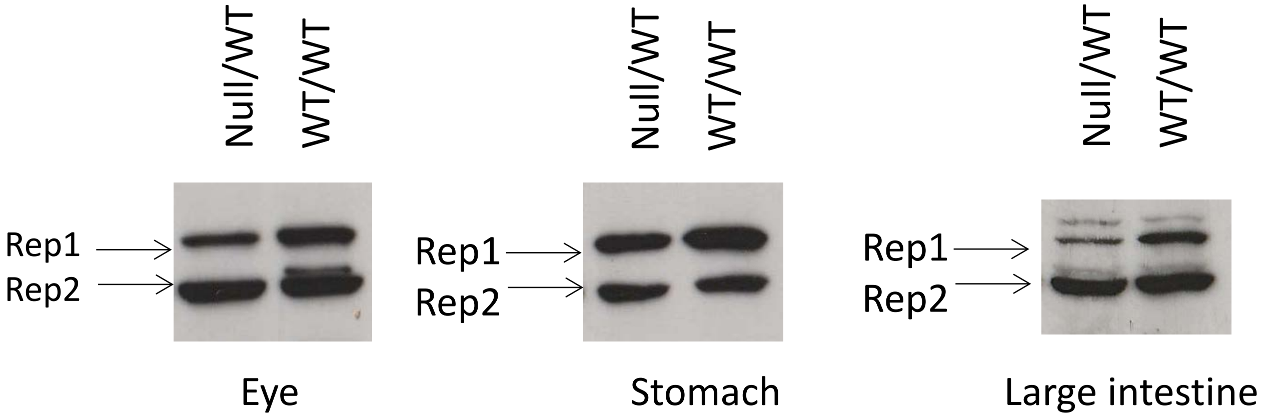

S1 Figure. **Rep1 expression in tissues of *CHM*$^{null/WT}$ females is reduced in comparison to the wild type mice.** Protein extracts were isolated from mouse tissues indicated underneath each blot (n=1), subjected to SDS-PAGE and analysed by immunoblotting using anti-Rep antibody (J905) which recognises both Rep1 and Rep2 (dilution 1:500). Age of animals: 8-9 months. Rep2 band was used as a loading control. Experiment was repeated 3 times.
