# Supplementary material for: Chronically shortened rod outer segments accompany photoreceptor cell death in Choroideremia
Source: PLoS One. 2020 Nov 17;15(11):e0242284. doi: 10.1371/journal.pone.0242284 (PMC7671558; doi:10.1371/journal.pone.0242284)
Supplement: S2 Fig — In vitro prenylation reaction was performed on the cytosolic lysates isolated from the neuroretina and RPE of the CHMWT/WT (n = 4), CHMFlox/Y (n = 5), TM-induced CHM3lox/Y, MerCreMer (MCM) (n = 3) and CHMnull/WT (n = 5) mice. CHMFlox is a conditional allele with level of Rep1 similar to CHMWT. CHM3lox is a conditional hypomorphic allele of CHM gene. TM induction of CHM3lox/Y, MCM leads to CHM knockout. Age of animals: 6–8 weeks. (PDF) [file pone.0242284.s003.pdf]

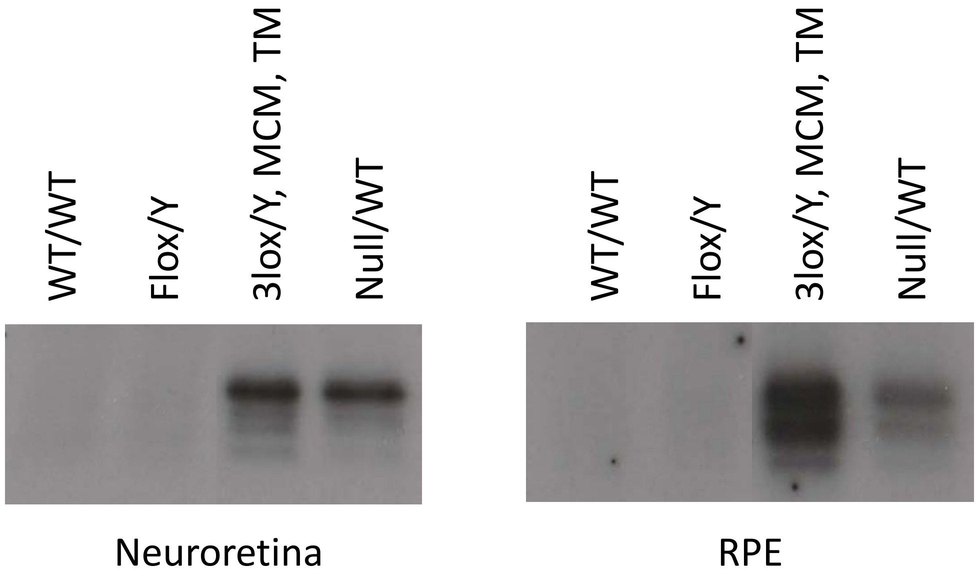

S2 Figure. Pattern of underprenylated Rabs in *CHM*$^{null/WT}$ is similar to tamoxifen(TM)-induced *CHM* knockout, yet amount of newly prenylated Rabs in *CHM*$^{null/WT}$ is lower, which is consistent with only 50% of cells carrying null allele. *In vitro* prenylation reaction was performed on the cytosolic lysates isolated from the neuroretina and RPE of the *CHM*$^{WT/WT}$ (n=4), *CHM*^{Flox}*/Y* (n=5), TM-induced *CHM*^{3lox}*/Y, MerCreMer (MCM)* (n=3) and *CHM*$^{null/WT}$ (n=5) mice. *CHM*^{Flox} is a conditional allele with level of Rep1 similar to *CHM*^{WT}. *CHM*^{3lox} is a conditional hypomorphic allele of *CHM* gene. TM induction of *CHM*^{3lox}*/Y, MCM* leads to *CHM* knockout. Age of animals: 6-8 weeks.
